# State of the Globe: Yellow Fever is Still Around and Active!

**DOI:** 10.4103/0974-777X.52975

**Published:** 2009

**Authors:** Dilip Mathai, A George Vasanthan

**Affiliations:** *Department of Internal Medicine, Training and Research Center, Christian Medical College, Vellore - 632 004, India.*; 1 *Department of Infectious Diseases, Training and Research Center, Christian Medical College, Vellore - 632 004, India.*

We have conquered space in the 21^st^ century but people are still dying from yellow fever. We know what causes the disease—a virus belonging to the family Flaviviridae. Although an effective vaccine is available, the WHO still estimates an annual occurrence of 200000 yellow fever cases with 30000 related deaths. These statistics are even more alarming when we consider the fact that no disease can be considered as strictly endemic today. Airplanes unwittingly transport infected travelers, vectors, viruses, bacteria, and their disease to all corners of the earth. Mosquitoes can carry the YF virus globally with the present ease in international travel. Americans, Europeans, and especially, Asians are vulnerable as they have the competent vector mosquitoes and nonhuman primates.[1]

Mosquito-borne Yellow fever (YF) can be considered as a typical example of an acute zoonotic hemorrhagic illness that has the potential to cause a worldwide pandemic. Although it is endemic in 33 African and 11 South American countries, actual areas of YF activity may exceed the officially reported zones.

It is estimated by unconfirmed reports that over a million people may visit regions endemic with yellow fever. Susceptible unimmunized forestry, industrial, and agricultural workers are infected following the bite of the vectors of Aedes spp. in Africa, and several species of Haemogogus in South America. These forest vectors survive by feeding on infected nonhuman primates in the jungle and then transmit the virus to humans (sylvatic or forest cycle). Viremic humans returning from forests, have the potential to transmit the virus to other humans through the domestic vector mosquito, Aedes aegypti (urban cycle).

Since the 1940s, the American tropics were at the highest risk for urban YF epidemics. Today, high primary vaccination coverage, which is 70% in enzootic areas of Brazil, has reduced the number of cases in South America to 10% of global occurrence. This is in stark contrast to Africa where 90% of the cases occur. The annual number of confirmed cases which is about 300 per year in S. America, may be an underreported underestimate of the true infective scenario. Unfortunately, the disease occurs in remote areas where recognition of outbreaks is delayed and diagnostic facilities to confirm the disease are absent or inadequate.

There has been a resurgence of YF in the last decade in large African and South American urban populations. This is due to an increase in the distribution and density of Aedes aegyptii as a result of deforestation, global warming, urbanization, human travel, and poor sanitation. An infected mosquito remains infectious for its lifetime of two to three months, and the virus can survive from season to season in its eggs. These mosquitoes bite during daytime hours so bed nets may not be protective. Most importantly, immunization cover in the susceptible population has been poor.

The study reported by Ricardo *et al*. in this issue of the Journal of Global Infectious Diseases (June 2009) estimated the risk in a forest transmission model of YF among military personnel in Ecuadorian Amazon. This was assessed by using the field monitoring tool of screening cases with elevated hepatic enzymes and confirmed by seroepidemiology. The jungle model of transmission due to deforestation (associated as risk factor) and the resulting increase in vector density in the detachments and outposts, resulting in disease transmission in an unvaccinated population, warrants that vaccination be made a high priority as there is no specific antiviral treatment. Improvements in intensive care have not changed the case fatality rate (CFR) due to YF. Efforts, therefore, should be made to discover antiviral drugs for flavivirus, not only for hepatitis C and dengue, but also YF.

The clinical presentation with an acute onset of jaundice and raised liver enzymes can mimic other more commonly occurring vector-borne, infectious diseases such as malaria, hemorrhagic viral fevers, dengue, rickettsial diseases, and food/water-borne diseases such as leptospirosis, typhoid fever, viral hepatitis (HEV), or acute fatty liver in pregnancy and poisoning (e.g., carbon tetrachloride). Investigators should be commended for a high degree of clinical suspicion and for robust investigations despite difficulties encountered in confirming the diagnosis. This discovery along with vaccination programs, has led to the conclusion of the outbreak within two months.

Diagnosis of these illnesses is seldom confirmed in these areas. This suggests a need for a greater awareness among the physicians, both in endemic patients and particularly, returning travelers from such areas. Clinical presentation of yellow fever are manifestations of the disease complications like thrombocytopenia, hemorrhaging, disseminated intravascular coagulation, massive liver necrosis, and fulminant hepatitis. Physicians should have a high index of suspicion and consider YF as a possibility in cases of hepato—renal syndrome, pulmonary edema, myocarditis, cardiac arrhythmias, and intracranial hemorrhage that can cause death. Mass vaccination campaigns should be initiated as a response to clinically suspected cases, a laboratory-confirmed human outbreak, or to reports of monkeys dying with the YF virus.

The Amazon study on YF risk has shown the CFR to be 6.8%. This low CFR seen among unimmunized soldiers infected in South America could be attributed to the role of an anamnestic immune response due to previous exposure to less lethal flavivirus infections such as dengue, and through better case management strategies with military assistance.

Rapid point-of-care tests have not yet been developed, but this will be critical when an antiviral agent is available. Demonstration of YF-specific IgM, and/or a fourfold or greater rise in serum IgG levels by monoclonal enzyme immunoassays (acute or convalescent), in the absence of recent YF vaccination, is sensitive and specific. Detection of YF antigen in serum or tissues by immunohistochemistry, or in situ hybridization with 35S-labeled negative chain RNA probes, viral genome sequences in blood, or other body fluid or tissues using RT-PCR are some other tests. Molecular tests for rapid and specific detection of YF in tissue samples of nonhuman primates are an important tool for epidemiologic surveillance. Isolation of YF virus in suckling mice and via cell culture in Vero cells or MOS 61 cells [Aedes pseudoscutellaris cell line] or C6/36 A. albopictus cells (the latter insect cell lines have the advantage of demonstrating a clear cytopathic effect) is the choice for diagnosis. Rapid virus identification will permit the timely activation of control systems for prevention of further cases and epidemic situations.

This clinical and seroepidemiological study has used virus isolation by conventional cell culture and mouse brain inoculation methods, followed by IF. Serum IgM Mac-ELISA and IgG ELISA were used at the nearest US regional medical research institute for the confirmation of clinical cases. An antibody titer of 200 was declared positive though no repeat tests were done to demonstrate a four-fold rise in titer. It was not deemed necessary in this case as all soldiers who were infected with YF virus, had not been previously immunized. A cross-reactive flaviviral antibody, such as dengue, is a challenge in diagnosis. However, the study in Amazon has ruled this out by using various cross-reacting antigens to YF virus as control and also as appropriate negative controls. This study had not done a confirmatory RT-PCR that is available at the WHO regional reference laboratory for the YF virus. It is known that daignosis of arboviral infections like yellow fever is done by serum or cerebrospinal fluid (CSF) analysis for virus-specific IgM and neutralizing antibodies.[2]

If these infected soldiers (in the viremic phase) had traveled to their urban homes where *A. aegypti* might exist, there would have been disastrous civilian infections. Also significant is the observation that bed nets were not protective, which suggests an exposure to vector bites occurring in the daytime. Army personnel, travelers, and infants below six months of age in the YF endemic zone would need to be immunized and active seroprevalence of adult civilians checked out during epidemics to assess their immune status.

YF virus is an enveloped, single-stranded, positive sense RNA virus. Despite several different genotypes, there is a single serotype that is antigenically conserved. Therefore, the 17D YF vaccine protects against all strains of the virus. YF can be substantially controlled through a single dose (unlike the avian and swine influenza which show antigenic drift).

One out of seven persons infected with the YF virus develops illness during an epidemic, but only one adverse event occurs for every 200,000 to 400,000 immunized with the YF vaccine. The WHO's International Health Regulations (2005) recommend that all individuals should obtain an International Certification of Vaccination or Prophylaxis (ICVP) if visiting endemic zones (or even in transit) and during outbreaks.[3]

The live-attenuated strain of YF virus known as 17D, has been used creating a Yellow Fever vaccine used in humans since more than 50 years.[4] The Global Advisory Committee on Vaccine Safety (GACVS) has made recommendations on the YF wild virus live vaccine (Asibi strain) in use today, which is an attenuated 17D variant vaccine and is considered as one of the least safe vaccines, following the recognition of associated viscerotropic disease (the subtypes are nontransmissible by mosquitoes).[5] Recent studies on pressure-inactivated virus showed that this vaccine elicited low levels of neutralizing antibody titers although it exhibited complete protection against an otherwise lethal challenge with 17D virus in the murine model. There is a need for the development of new inactivated yellow fever vaccines for travelers to areas or countries with risk of YF transmission. The use of a full-length cDNA clone of 17D-204 virus is in the pipeline for manufacturing the live 17D vaccine. This new method allows production in a cell culture system and hence, reduces the risk of adventitious viruses and the selection of a subpopulation during replication, and increases safety.

The live vaccine dose given subcutaneously has a highly favorable benefit-risk profile (with 99.9% protection within a month) and sustains immunity for over 35 years (ten years is the rule for revaccination). A single vaccination to cover 80% of the susceptible population is recommended for protection. Children should be immunized at nine months through EPI in YF endemic countries. However, at present, the stockpile of vaccine available at the WHO may not be adequate if a large outbreak occurs. Newer vaccine preparations are envisaged and safe vaccines need to be developed for use in pregnant women and those below six months of age.

Over the last 20 years, the numbers of YF epidemics have risen and more countries are reporting cases. There is still a large susceptible, unvaccinated population in both Africa and the Americas. Changes in the world's environment, such as deforestation, global warming, and urbanization, have increased mosquito numbers and habitats, enabling frequent contact of humans, mosquito, and virus. It is true that YF remains the only disease for which proof of vaccination is required as a condition on entry. Widespread international travel could play a role in spreading the disease, an event that may constitute a “public health emergency of international concern”. Therefore, mass vaccination campaigns and surveillance should continue.
